# Evidence-based fluid resuscitation of the septic HFpEF patient: A narrative review of the literature

**DOI:** 10.2478/jccm-2026-0005

**Published:** 2026-01-30

**Authors:** Maxwell Ward, Roshan Acharya, Anthony Loschner

**Affiliations:** Virginia Polytechnic Institute and State University, Carilion School of Medicine, Roanoke, Virginia, USA

**Keywords:** HFpEF, sepsis, fluids, resuscitation, shock, heart failure

## Abstract

**Purpose:**

This narrative review aims to highlight the available evidence on fluid resuscitation in septic patients with heart failure, with a particular focus on heart failure with preserved ejection fraction.

**Methods:**

A PubMed search was conducted using the keywords “sepsis” (or sepsis, or septic shock), “heart failure” (or HF, or HFrEF, or HFpEF or congestive heart failure), and “fluid” (or resuscitation, or fluid resuscitation, or fluid management). The results were summarized in narrative review format.

**Results/Conclusions:**

The presence of HFpEF in septic patients appears to be associated with an increased risk of adverse outcomes. This population may benefit from a more individualized approach to fluid resuscitation. Emerging tools for assessing fluid responsiveness and characterizing septic cardiovascular physiology show promise, but further investigation is needed.

## Introduction

The 30 cc/kg intravenous fluid bolus is recommended as the appropriate initial approach to volume resuscitation in patients with severe sepsis or septic shock. A review of current literature suggests variability in the way clinicians view this recommendation, especially regarding septic patients with concomitant heart failure (HF). Interestingly, the surviving sepsis campaign downgraded the strength of their recommendation on this topic due to weak evidence [1]. While there are existing studies that address fluid resuscitation of septic congestive heart failure patients, there is a paucity of review articles that summarize the current evidence. Furthermore, fluid resuscitation of septic heart failure patients with preserved ejection fraction (EF) remains a topic in need of greater investigation and guidance. We performed this narrative review to appraise the recent studies investigating the 30 cc/kg fluid bolus adherence and outcomes in septic patients with heart failure, with a special focus on HFpEF. This review begins with a comprehensive evaluation of clinical guidelines and real-world adherence for the management of fluid in septic patients with concomitant heart failure. Building on this framework, the narrative then transitions into a deeper analysis contrasting outcomes and evidence specifically for patients with heart failure with preserved EF (HFpEF) versus those with heart failure with reduced EF (HFrEF).

## Methods

A PubMed search was conducted (on 12/11/2024) using the terms sepsis (or sepsis, or septic shock), heart failure (or HF, or HFrEF, or HFpEF or congestive heart failure), and fluid (or resuscitation, or fluid resuscitation, or fluid management). Filters for papers published since 2000 and English language were applied. Individual review of resulting abstracts was performed to screen out articles which were found to be irrelevant to the topic or met exclusion criteria. Exclusion criteria: pediatric studies, retracted articles, studies coincidentally mentioning all keywords but without relation to the topic. Referenced papers were included if they provided further context to a topic and did not meet exclusion criteria. 30 papers were selected, and each paper underwent a thorough review process whereby the study type, primary findings, strengths, and criticisms were summarized in narrative format (Supplementary material).

## Results

### Current guidelines

The most recent guidelines published by the SCCM surviving sepsis campaign recommend a 30 cc/kg bolus of intravenous crystalloid within 3 hours for all-comers with severe sepsis or septic shock. This blanket recommendation has received criticism with more evidence emerging that standardization of an early fluid bolus does not meaningfully affect outcomes in patients with sepsis [2,3,4]. Additionally, this guideline does not differentiate between patients with concomitant volume-overloading processes such as HF, cirrhosis, or end-stage renal disease. This lack of individualization raises the question of whether such patients require a different approach.

### Adherence to guidelines

Our literature review revealed four recent studies that directly evaluated clinician use of IV fluids in septic HF patients as a primary outcome. Three of the studies [5,6,7] measured the amount of fluid given to patients with sepsis, and all three studies concluded that heart failure patients (HFrEF specifically in one study) receive significantly less crystalloid. The fourth study [8], a 2017 prospective cohort study, measured timeliness of fluid resuscitation and found that the presence of heart failure and renal failure significantly delayed fluid initiation. While there is a trend in practice towards the recommended 30 cc/kg bolus within the first 3 hours among all-comers, our review elucidates that clinicians are less likely to meet this goal (both in fluid volume delivered and timeliness) when patients present with heart failure. The more salient question is whether this hesitation is warranted.

### Outcomes in septic heart failure patients

We found fifteen studies measuring clinical outcomes in septic HF patients. Each of these studies were designed with a different intent, and there was a mixed bag of findings. As expected, the presence of heart failure as a comorbidity generally tends to increase mortality and/or adverse outcomes such as intubation and steroid use in septic patients [9,10].

One observational study by Truong et al. looked at patients with septic shock and assessed if compliance with a 30 cc/kg protocol is associated with a difference in outcomes [11]. Firstly, Truong et al. corroborated the conclusion that clinicians tend to give less fluids in heart failure patients. Additionally, this study provided more insight on whether the protocol leads to improved outcomes when adhered to. When they compared covariate-matched septic shock patients who received the protocolized fluid to those who didn’t, they found no significant difference in in-hospital mortality. While this data does not guide us in the use of fluids for the septic HF patient in particular, it does introduce the idea that an individualized approach to fluid volume administration may be acceptable.

A 2020 retrospective cohort study by Khan et al. studied high-risk patients (those with HF, cirrhosis, and ESRD) with sepsis [12]. They set out to determine if protocolized fluid administration (30cc/kg within 6 hours) affected rates of mechanical ventilation at 72 hours and concluded that there was no difference in this outcome. It introduced the potential safety of aggressive initial fluid resuscitation in the aforementioned high-risk groups. A 2022 systematic review and meta-analysis supported this finding, demonstrating no increase in adverse events from receiving the recommended bolus amongst HF and ESRD patients with septic shock [12]. Furthermore, Acharya et al. [7] found an inverse correlation with fluid administration and in-hospital mortality in septic HF patients, demonstrating a 12% mortality reduction with each 250 ml of fluid given within the first 6 hours for this population. They demonstrated no significant increase in mechanical ventilation in CHF patients with severe sepsis or septic shock receiving ≥30 cc/kg. The study by Acharya et al. included HF with severe sepsis and septic shock exclusively. A 2023 systematic review and meta-analysis by Vaeli Zhadeh et al [13] incorporated 4 research studies to reach a supporting conclusion; that a restricted volume approach (<30 cc/kg within 3h) was associated with higher in-hospital mortality. Outcomes based on fluid resuscitation in these high-risk patients continues to be a topic of active research [15]. Not all of the resulting studies argued for more fluids in HF patients, however.

Al Abassi et. al. found that the implementation of Surviving Sepsis Campaign guidelines leads to a more aggressive administration of fluids in women with HF than in men [20]. While there were no significant mortality differences between men and women, some subgroups of women experienced higher rates of flash pulmonary edema as an example. Further delineation of these subgroups is explored in subsection 5.

A unique 2024 retrospective study [16] assessed outcomes in patients with sepsis and acute decompensated heart failure. Researchers Weng and Xu stratified patients into distinct categories based on volume of fluid received per ideal body weight (cc/kg). They found an optimal target of 10–15 cc/kg within the first 3 hours demonstrated improved in-hospital mortality compared to volumes of >20 cc/kg or <10 cc/kg. They also found that resuscitation volumes exceeding 20 cc/kg were associated with significantly higher rates of endotracheal intubation. Their findings can be interpreted to validate the role of clinical volume assessment and isolate acute decompensated HF from simply a history of HF.

A 2024 retrospective cohort study [17] investigated fluid administration within the first 6 hours in septic HF patients without volume overload on presentation. In contrast to Acharya and Vaeli Zadeh, Beagle et al. found a near-linear correlation between volume of resuscitation and a composite of in-hospital mortality and discharge to hospice.

### Timeliness of fluid initiation in septic heart failure patients

An important topic in our review of the literature was the timeliness of fluid administration. One study sought to determine the relationship of fluid initiation timing with outcomes. Leisman et al. found that crystalloid resuscitation started within 2 hours was associated with improved mortality, mechanical ventilation, ICU need, and length of stay [8]. Importantly, these associations were maintained within the CHF subgroup. Kuttab et al. added to this body of evidence in 2021, when they found that failure to complete 30 cc/kg within 3 hours was associated with increased odds of mortality, delayed hypotension, and increased ICU length of stay [18]. Acharya et al. found that only 39% of patients with CHF received 30 cc/kg fluid bolus within 6 hours [7].

### HFpEF vs HFrEF in sepsis

The syndrome of congestive heart failure (CHF) encompasses a heterogenous set of phenotypes. Too often in the clinical setting, the presence of reduced ejection fraction is conflated with HF which risks underestimating the clinical detriment of congestion in those with preserved EF. Furthermore, when HFrEF and HFpEF are grouped under the umbrella of HF, it risks aggregation bias. In relation to the rising prevalence of HFpEF worldwide, the amount of clinical research aimed at guiding fluid resuscitation in septic HFpEF patients (rather than patients with HF in general) is severely lacking. Unfortunately, none of the studies in our search directly compared outcomes in septic HFpEF vs septic HFrEF patients as a function of volume resuscitation. This remains an area of research in need of further investigation. Our literature search did, however, yield several studies with findings that can be used to draw conclusions in septic patients on the basis of left ventricular (LV) function. We identified five key studies that evaluated for an association between left ventricular function (LVF), typically via EF, and adherence to fluid management guidelines (Table 1).

**Table 1.** Association between left ventricular dysfunction and fluid management

**Author (Year)**	**Objective**	**Key Findings**	**Did LV Dysfunction Predict Adherence to Fluid Bolus?**
Franco Palacios CR et al. (2019)	Identify factors that affect fluid resuscitation in septic patients [5]	-LV function (EF or diastolic dysfunction) did not predict fluid volume administered, despite HF history being associated with less fluid given	No

Al Abbasi et al. (2020)	Assess gender-specific compliance with >30 mL/Kg fluid bolus and outcomes in patients with CHF [20]	-Women with HFpEF received higher volumes of fluid than men with HFpEF	No^*^
		-Men and women with HFrEF received similar amounts of fluid	

Acharya et al. (2021)	Assess compliance with >30 mL/Kg fluid bolus in septic patients with and without CHF and their outcomes [7]	-The presence of a reduced EF did not affect the chances of getting a fluid bolus.	No

Ehrman et al. (2022)	Assess the association between volume of IV crystalloid and outcomes in septic patients with reduced LVEF [19]	-HFrEF patients received similar IVF at 2h compared to preserved EF	No

Powell et al. (2022)	Assess compliance with >30 mL/kg bolus in septic HFrEF patients [6]	-HFrEF patients were less likely to receive IVF target at 6h compared to preserved EF	Yes

* While EF alone did not predict fluid administration in this study, EF combined with gender did.

With exception of one study [6], we found that EF and diastolic function did not independently predict the volume of fluid patients received [5,7,19,20]. When taken into context with the knowledge that presence of CHF impacts clinician use of fluids [5,6,7,8], it suggests that clinicians base fluid resuscitation volumes on clinical history rather than echocardiographic parameters. Investigating for an association between left ventricular function and outcomes in septic heart failure patients, our search yielded six impactful studies (Table 2).

**Table 2.** Association between left ventricular function and patient outcomes

**Author (Year)**	**Objective**	**Measure of LV Function**	**Did LV Dysfunction Predict Outcomes?**
Palmieri et al. (2015)	Assessed the prognostic relevance of EF and global longitudinal LV systolic peak strain in sepsis [21]	EF	No
		GLS	Yes
Al Abbasi et al. (2020)	Assess gender-specific compliance with >30 mL/Kg fluid bolus and outcomes in patients with CHF [20]	EF	No^*^
Hai et al. (2020)	Evaluate the prognostic value of a LV systolic function using speckle tracking echocardiography in patients with septic shock [22]	Conventional 2D echo measures (EF, LVEDV, etc.)	No
		GLS	Yes
Khan et al. (2020)	Assess the association between 30 mL/kg bolus and intubation in patients with sepsis or septic shock and HF, ESRD, or cirrhosis [12]	EF	No
Acharya et al. (2021)	Assess compliance with >30 mL/Kg fluid bolus in septic patients with and without CHF and their outcomes [7]	EF	Yes
Ehrman et al. (2022)	Assess the association between volume of IV crystalloid and outcomes in septic patients with reduced LVEF [19]	EF	No
Weng et al. (2024)	Assess the impact of early fluid dosing in septic patients with acute decompensated heart failure [16]	EF	Yes^**^

* While EF alone did not predict outcomes in this study, EF combined with gender did;

** HFrEF patients were not directly compared to HFpEF in this study’s analysis, however, an optimal fluid strategy of 10–15 cc/kg was seen in the HFrEF subgroup and not the HFpEF subgroup;

GLS = global longitudinal left ventricular systolic peak strain.

Ejection fraction measured by traditional echocardiography was not always predictive of outcomes [19,20,21,22]. Chebl et al. demonstrated that HFpEF combined with echocardiographic diastolic dysfunction had higher rates of ED mortality, intubation, and steroid use than non-CHF patients [10]. One study revealed a lower ideal 3h fluid target (10–15 cc/kg) in reduced EF patients with acute decompensated HF and sepsis, however, it did not directly compare HFrEF and HFpEF patients [16]. Additionally, amongst the HFpEF population, there is evidence to suggest that women receive higher relative fluid volumes and experience more pulmonary edema, whereas men of this subgroup experience more cardiogenic shock. Lastly, two studies demonstrated that measurement of GLS was superior to EF in predicting short-term mortality amongst septic patients [21,22].

An important takeaway from these findings is that EF alone has not been shown to reliably predict outcomes in septic patients. Gender, diastolic dysfunction, and presence of acute decompensation are factors that, in combination with EF, hold more predictive value. GLS outperforms conventional echocardiographic measures in this area as well. Further research is needed to determine optimal fluid strategies in septic HFpEF patients with direct comparison to septic HFrEF patients.

### Evidence Based Parameters and Monitoring Response to Therapy

B-type natriuretic peptide (BNP) levels measured in septic patients on admission may provide prognostic value regarding in-hospital mortality in septic patients [23]. Our search did not reveal a further known utility in guidance of fluid therapy for BNP. Global longitudinal strain measured by speckle tracking also offers prognostic value but has not yet been used to guide dynamic fluid resuscitation [21,22]. Left ventricular ejection fraction (LVEF) measured at ED presentation did not independently increase risk of adverse outcomes in one small prospective cohort study of septic patients [19]; whereas in another study, a history of EF <40% was associated with 2.7-fold increase in mortality [7]. In the case of septic HFpEF patients, lactic acid at presentation is positively correlated with higher in-hospital mortality [10].

Fluid accumulation index (FAI) is a metric which compares fluid balance to fluid intake (FB/FI). FAI may serve as an important guiding parameter for fluid resuscitation in heart failure patients as high FAI within the first 48 hours is associated with increased in-hospital mortality in septic HF patients [24]. While our search did not reveal any prospective trials on FAI in septic HF patients, FAI is a promising tool. Dong et al. demonstrated an association of high FAI with mortality, whereas FI and FB were not [24]. A positive FB among non-critically ill patients at discharge is not correlated with hospital readmission risk [25], however, more investigation is needed in the critically ill population.

Emerging evidence suggests that transesophageal echocardiogram is capable of categorizing septic patients into distinct phenotypes with regard to their cardiac response to septic shock [26]. In non-CHF patients, septic shock can produce an array of echocardiographic and clinical parameters that fall into predictable clusters such as “well-resuscitated, LV systolic dysfunction, hyperkinetic profile, RV failure, and sustain hypovolemia” groups [26]. Importantly, this has not yet been applied to heart failure patients and requires more investigation. The variability in physiology among septic patients is a complex dilemma for clinicians, particularly in HF and pulmonary hypertension patients who may require individualized therapy for optimization of cardiac output [27].

The E/e’ ratio is an echocardiographic index which is typically applied in the diagnosis of diastolic dysfunction. A threshold value of 14 has been widely used in cardiology to assess for elevated filling pressures in the left ventricle. Krantz et. al contend that E/e’ may be a clinically significant indicator when deciding on fluid resuscitation vs diuresis in unexplained dyspneic patients with a presentation concerning for pulmonary sepsis versus cardiogenic edema [28]. Use of the E/e’ in this setting is, however, has not been studied rigorously and requires further exploration.

Left ventricular outflow tract (LVOT) velocity is another echocardiographic measurement that has been hypothesized to guide fluid management. Chiem and Turner propose a two step point-of-care ultrasound technique to calculate a change in velocity time integral (ΔVTI) or change in maximal velocity (ΔVmax) across the aortic valve [20]. These two parameters are suggested to be predictors of fluid responsiveness that can serve as an alternative to caval sonography which has known limitations (ie. intubated patients, difficult windows).

## Discussion

Our review of the literature supports an emphasis on timeliness of initial fluid resuscitation in septic heart failure patients [8,18]. Initiation of fluids should not be delayed, and further examination and history obtainment should be promptly undertaken while isotonic fluids are being delivered. A history of heart failure, admitting BNP >500 pg/mL, and LV systolic dysfunction measured by GLS serve as negative prognostic indicators regarding mortality in septic patients [9,21,22,23]. Presence of HFpEF may not increase mortality but likely predicts an increase in adverse outcomes. The evidence to implicate guideline-correspondent fluid resuscitation as a source of these adverse outcomes is lacking and remains a controversy. However, women with HF may be an exception, as clinicians are prone to over-resuscitating this population [20]. Men and women with sepsis and HF appear to differ in terms of adverse outcomes [20], and clinicians should be aware of these sex-based differences to anticipate adverse events. Additionally, it is important to distinguish septic patients with simply a history of HF from septic patients with acute decompensated HF as the latter group may require a fluid volume target in the 10–15 cc/kg range [16]. Our review focused primarily on fluid resuscitation. Addition, timing, and titration of vasopressors was not thoroughly explored in our review, but we recognize the importance of defining their role in the treatment of septic HF patients.

Dynamic measures should be employed to monitor the patient’s response to therapy. Emerging evidence suggests that defining the patient’s septic cardiac phenotype and monitoring FAI may serve as tools to achieve optimal cardiac output [26]. Echocardiographic measurements of E/e’ ratio and dynamic LVOT velocity have been hypothesized to guide fluid decisions beneficially [28,29], but require further investigation. Lastly, the presence of concomitant cardiogenic shock should be carefully assessed, as temporary inotropic and/or mechanical circulatory support may be indicated [30].

Looking forward, more research is needed to guide fluid management in septic patients with HFpEF. Certainly, prospective studies comparing outcomes in septic HFrEF vs septic HFpEF patients stratified to different fluid strategies would be of great value. If future research continues to show ambiguity in the prognostic utility of EF in the septic HF population, we purport that a shift in thinking is warranted. Focusing on GLS, sex, and acute decompensation to individualistically identify a patient’s phenotype may be prudent [20,21,22]. To guide ongoing fluid resuscitation, studies involving dynamic response to therapy such as FAI, E/e’ ratio, dynamic LVOT velocity should be upscaled to solidify our understanding of their role [26,28,29].

As the prevalence of HFpEF increases, clinicians will continue to be faced with challenging fluid management decisions. In all-comers with sepsis, we recommend timely initial volume resuscitation followed by prompt clinical volume assessment to determine presence of acute decompensated heart failure. History of heart failure should be elicited including thorough chart review. If available, expedited echocardiography should be performed to establish a baseline EF, GLS, E/e’ ratio, and LVOT velocity. Women with sepsis and HFpEF should be thought of as high-risk for pulmonary edema and warrant a higher index of suspicion for this complication if their respiratory status worsens. Accurate in-and-out charting is essential. Clinicians should consider calculating FAI at 48 hours for further prognostication, as as a ratio of >0.42 at this juncture is associated with higher in-hospital mortality.

## Conclusion

The 30 cc/kg bolus has been adopted as the gold standard fluid strategy for all-comers with severe sepsis or septic shock which often causes concern for fluid overload in heart failure patients. The presence of HFpEF likely predicts worse outcomes and may warrant an individualized approach to fluid resuscitation. Dynamic and static measures of fluid responsiveness are promising tools to guide fluid therapy in patients with a perceived risk for fluid overload.

## Abbreviations

CHF: congestive heart failure
EF: ejection fraction
GLS: global longitudinal left ventricular systolic peak strain
HF: heart failure
HFpEF: heart failure with preserved ejection fraction
HFrEF: heart failure with reduced ejection fraction
IV: intravenous
IVF: intravenous fluid(s)
LV: left ventricular or left ventricle
LVF: left ventricular function
